# Synthesis of fly ash-based zeolite and experimentation in catalytic converter for NO_x_ reduction on diesel engines: a comparative study

**DOI:** 10.1038/s41598-026-51800-8

**Published:** 2026-05-20

**Authors:** P. Rajakrishnamoorthy, M. Bakkiyaraj, Robert Cep, B. Swarna

**Affiliations:** 1 Department of Mechanical Engineering, Meenakshi Ramaswamy Engineering College, Ariyalur, 621804 India; 2 Department of Mechanical Engineering, Rajalakshmi Institute of Technology, Chennai, 600124 India; 3 Department of Machining, Assembly and Engineering Metrology, Faculty of Mechanical Engineering, VSB-Technical University of Ostrava, 70800 Ostrava, Czech Republic; 4 Department of Biosciences, Saveetha School of Engineering, Saveetha Institute of Medical and Technical Sciences, Chennai, 602105 India; 5 University Centre for Research & Development, Chandigarh University, Mohali, 140413 India

**Keywords:** Fly ash, Zsm5-zeolite, Wash coating, CI engine, NO_x_ reduction, Chemistry, Energy science and technology, Engineering, Environmental sciences, Materials science

## Abstract

This study presents the synthesis of low-cost ZSM-5 zeolite-like materials from hazardous coal fly ash and their application as metal-doped catalysts for nitrogen oxides (NO_x_) reduction in diesel engine exhaust. The synthesized zeolite was prepared through acid treatment and hydrothermal crystallization, followed by metal doping with Cu, Fe, and Ce using an ion-exchange method. The catalysts were characterized using SEM, XRF, and XRD. Metal-doped zeolite formulations (Fe-ZSM-5, Cu-ZSM-5, Fe–Ce-ZSM-5, and Cu–Ce-ZSM-5,) were wash-coated onto cordierite monoliths and tested in a four-stroke CRDI diesel engine. The performance of the self-made catalytic converters (CCs) was compared with a commercial catalytic converter (CCC). Results showed that Fe–Ce-ZSM-5 and Cu–Ce-ZSM-5 achieved NO_x_ reductions of 69% and 71%, respectively, at full load, which were approximately 7% higher than that of the commercial CC. All developed catalysts demonstrated significant reductions in hydrocarbons (HC) and carbon monoxide (CO) emissions, with only minimal influence on brake thermal efficiency (BTE). The findings demonstrate that fly-ash–derived ZSM-5 is a promising sustainable catalyst support for diesel exhaust after-treatment applications.

## Introduction

Internal combustion engines (ICEs) are the most commonly used engines in the road transportation worldwide; nevertheless, they are one of the greatest sources of air pollution. Gasoline engines emits large amounts of CO, unburned HC, and NO_x_, while compression-ignition (CI) diesel engines also generate soot and particulate matter (PM). These emissions are known to cause a number of environmental and health challenges, including the development of ground-level ozone (smog), global warming and acid rain. NO_x_ and volatile organic compounds (VOCs) participates in photochemical reactions that have undesirable effects on the respiratory system of people. Specifically, Nitrous oxide (N_2_O) possesses a significantly higher global warming potential than CO_2_. In addition, nitrates and Nitrous dioxide (NO_2)_ contribute to reduced atmosphere visibility due to scattering of light. As transport sector reports, fossil-driven vehicles are account for more than 60% of the total anthropogenic emissions^1^.

The wide use of diesel engines in commercial goods vehicles, military transporters, and modern utility vehicles is attributed to their higher thermal efficiency, superior load carrying ability, and lower maintenance cost compared to gasoline engines. Nevertheless, diesel emissions have more toxic pollutants. The increasing the number of vehicles further worsening situation of air quality, posing a severe environmental threat^2^. In order to address the above concerns, stringent emission regulations are introduced periodically by the global pollution control authorities, typically every five years. The introduction of the Euro VI emission standards in 2020, which replaced the previous Euro IV standards, significantly restricted diesel engine emissions limits. These high standards have placed a considerable pressure on the automotive companies and researchers to come up with engines that comply with the required emission levels without compromising performance.

Despite the efforts made to minimize the emissions through the improvement in design of combustion, the mixing of air–fuel, and in-cylinder treatment methods, the process of reducing the emissions of the diesel exhaust continues to rely on further development of the after-treatment technologies^3^. These techniques include exhaust gas treatment systems, parametric optimization, absorber-based tuning techniques, and selective in-cylinder modifications. Nevertheless, traditional engine improvement methods have reached technological saturation, shifting research focus toward advanced catalytic post-treatment systems installed downstream of the engine^4,5^.

There is extensive application of cordierite honeycomb monoliths as substrates in diesel after-treatment systems due to their characteristics of low thermal expansion, high surface area, and structural stability. These monoliths are normally covered with precious metal catalysts, namely platinum (Pt), palladium (Pd), and rhodium (Rh) to oxidize CO and HC and to reduce NO_x_ emissions^6,7^. Diesel vehicles that meet the Euro VI requirements usually incorporate several after-treatment units arranged in series, all based on cordierite monolith architecture. Even though noble metal-based catalysts are effective, they are expensive and sensitive to sulphur compounds thus alternative low-cost materials need to be developed.

The zeolite-based catalysts of ZSM-5 have received more attention over the past years because of their distinctive microporous structure, high acidity, high thermal stability, and ability to perform desulfurization, denitrification, oxidation of hydrocarbons, and hydrocracking^8,9^. Numerous studies have been conducted on the zeolites synthesis using industrial wastes, particularly coal fly ash, which contains a high amount silica and alumina. ZSM-5-like materials have been synthesized through alkaline hydrothermal processes using fly ash and this has provided a viable way of recycling waste products while also lowering the cost of producing catalysts^10–15^. Nevertheless, few studies have successfully synthesized ZSM-5 using fly ash and tested it on real diesel engine exhaust systems. The majority of the available literature uses commercially obtained zeolites for emission control research.

This research investigates the development of a low-cost catalytic converter based on ZSM-5 zeolite prepared using coal fly ash. The prepared zeolite was modified with copper (Cu) and iron (Fe) to increase the redox activity, and further incorporated with cerium (Ce) to increase oxygen storage capacity and low temperature performance. Mono-metal (Cu-ZSM-5 and Fe-ZSM-5) and bimetal (Cu–Ce-ZSM-5 and Fe–Ce-ZSM-5) catalysts were made, wash-coated onto cordierite monolith substrates and tested in a diesel engine. The performance of these self-prepared catalytic converters were compared with that of an engine manufacturer catalytic converter (EMCC) which utilizes expensive noble metals like Pt and Pd.

## Materials and methods

### Synthesis and characterization of catalyst

The primary silica-alumina source in the synthesis of catalysts was coal fly ash which is a hazardous industrial by-product. The fly ash was purchased from Neyveli Lignite Corporation (NLC) in India. To avoid the absorption of moisture and any chemical change before the synthesis process, the fly ash collected was placed in airtight containers and then kept in a desiccator.

The X-ray fluorescence (XRF) analysis was utilized to estimate the elemental composition of the fly ash and their findings are shown in Table 1. According to the XRF data, the most prominent oxides are SiO_2_ and Al_2_O_3_ which together form about 79.30% of the total composition which is consistent with earlier reports^16–18^. Bivalent metal oxides like MgO and CaO are found in small amounts while monovalent oxides (Na_2_O, K_2_O) are added in traces. These are characteristic of Class-F fly ash and are also said to be capable of becoming good precursors for zeolites.

**Table 1:** Fly-ash chemical composition.

Elements	Al_2_O_3_	SiO_2_	Fe_2_O_3_	TiO_2_	MgO	CaO	SO_3_	K_2_O	P_2_O_5_	Na_2_O	BaO	LOi
Weight %	24.73	54.57	4.17	2.85	2.19	2.07	2.22	1.62	2.11	0.93	0.95	1.59

The previous research by Querol and colleagues^19^ has demonstrated that fly ash containing similar silica-alumina properties can be effectively transformed into zeolite-like structures, including faujasite and sodalite, because of its similarities with volcanic ash materials in hydrothermal synthesis^20–22^. The elevated Si/Al molar ratio in the XRF analysis is also highly beneficial in the current work in terms of obtaining ZSM-5-type zeolite which implies the use of a silica-rich precursor to develop the framework and achieve better catalytic activity in the reduction of NO_x_.

**Fig. 1:**
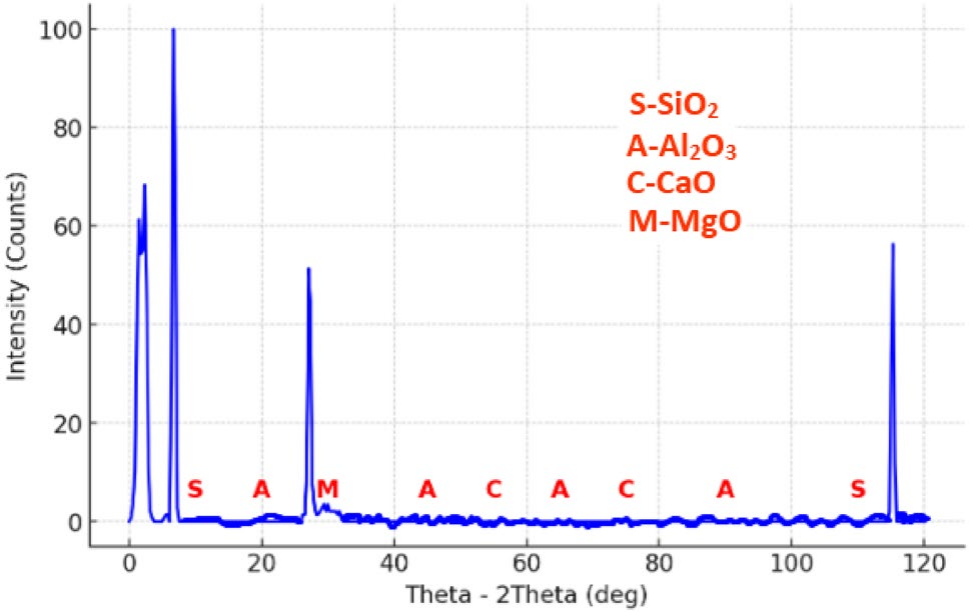
XRD pattern of fly ash.

X-ray diffraction (XRD) technique was employed to study the mineralogical phases that exist in the fly ash. The XRD pattern of the raw fly ash and the diffraction peaks corresponding to major crystalline phases such as SiO_2_ (quartz), Al_2_O_3_, CaO, and MgO are indicated using standard JCPDS diffraction peaks. The diffraction pattern indicates the typical quartz peak at 2 thetas = 26.6°, and other minor peaks corresponding to mullite, alumina and trace metal oxides. These crystal phases, together with the amorphous glassy fraction characteristic of fly ash, offer reactive sites that enable nucleation and crystallization of ZSM-5 in the hydrothermal synthesis process. It is also important to have amorphous aluminosilicate phases, which readily dissolve in alkaline conditions providing the required silicate and aluminate species to build the zeolite framework. Therefore, the XRF and XRD results prove that the collected fly ash has the necessary chemical composition and mineralogical properties to form the ZSM-5 zeolite-like materials (Fig. 1).

### Acid treatment of fly ash

**Fig. 2:**
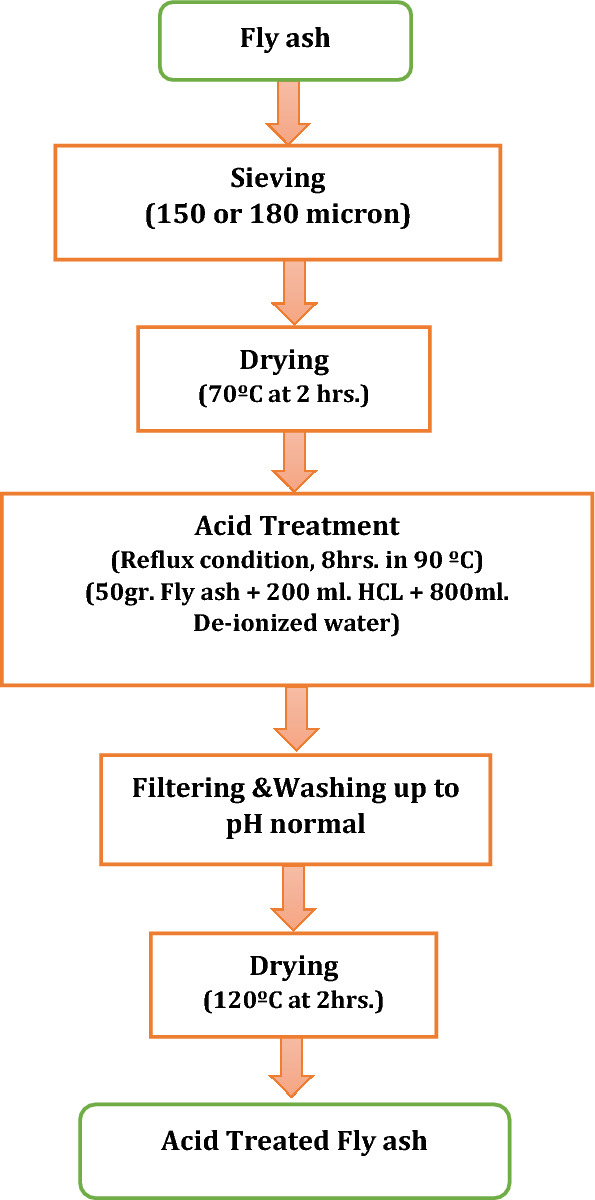
Flow diagram of acid treated fly ash.

An acid treatment process was employed following the process described by Missengue et al.^23^ to intensify the reactivity of the raw fly ash and eliminate unwanted impurities. Firstly, the fly ash was sieved to eliminate coarse and unreactive particles using a 180 µm sieve. The sieved fraction was washed with acid in a solution of 20% hydrochloric acid (HCl) and 80% deionized water.To treat the fly ash, 20 mL of the prepared acid solution was applied to every gram of fly ash. A reflux condenser was placed, and a mechanical stirrer was positioned overhead in the mixture in a round-bottom flask. The suspension was kept at 80 °C and stirred. Once the reflux treatment had been completed, the slurry was allowed to reach room temperature and subsequently filtered. The suspended solids were filtered again with deionized water and allowed to stir for 15 min to allow the fine ash particles to settle. The suspension was stirred with deionized water several times until the suspension was clear. The filtrate was filtered and washed repeatedly until the pH attained neutral, which means that the remaining acid and chloride ions were removed^24–27^. The flow chart of the acid treatment process of fly ash is depicted in Fig. 2.

### ZSM5 Zeolite preparation process

**Fig. 3:**
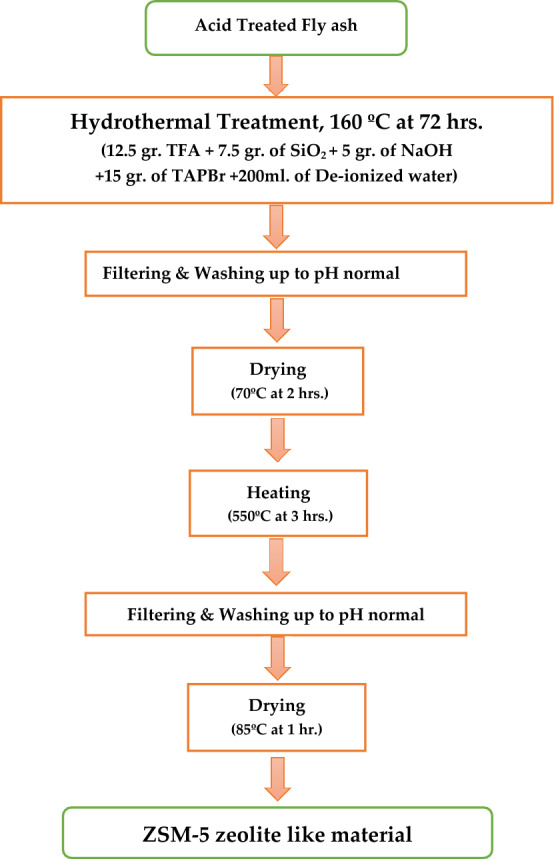
Conversion process of acid treated fly ash (TFA) into ZSM5 material.

The synthesis of ZSM-5 was performed after the acid treatment in accordance with a standard hydrothermal procedure. The fly ash (treated) and fumed silica (7.5 g and 12.5 g respectively) were added to 200 mL of deionized water, which was stirred. Sodium hydroxide (5 g) was added slowly to the mixture to ensure that it reached the desired level of alkalinity to dissolve the aluminosilicate species. Thereafter, the structure-directing agent, i.e., tetrapropylammonium bromide (TPABr) was added in a quality of 15 g quantities. The gel that was obtained was left to dry for one hour at room temperature and then placed into an autoclave lined with Teflon. The autoclave was placed in a box-type furnace and heated at 160 °C for 72 h to encourage the nucleation and crystallization of the ZSM-5 framework. Once the hydrothermal treatment was done, the solid product was filtered and washed with a large amount of deionized water. The dried material was dried at 70 °C and it took 2 h. To eliminate the organic template (TPABr), the product was calcined in air by heating to 550 °C with a ramp rate of 15° C/min and maintained at the final temperature of 550 °C, for 3 h. The dried product was rinsed with distilled water again until it reached a neutral pH and was dried thoroughly at 85° C for 1 h. The resultant product is a zeolite-like synthesized product using acid-treated fly ash (TFA)^28^. The entire conversion of TFA to ZSM-5 is depicted in Fig. 3.

### Zeolite based catalysts preparation

The synthesised ZSM5 zeolite material underwent doping with copper, iron, cerium-copper, and cerium-iron, using the Na + ion-exchange process. In this study, copper and iron were individually doped into ZSM5 zeolite, leading to the formation of monometallic zeolite products, specifically Cu-ZSM5 and Fe-ZSM-5. The remaining two sets consist of bimetallic ZSM-5 zeolite infused with Ce–Cu and Fe–Ce, resulting in Cu–Ce-ZSM5 and Fe–Ce-ZSM5.

#### Preparation of monometallic catalysts

The monometallic Cu-ZSM5 catalyst obtained by blending ZSM-5, and 0.5 M CuCl_2_ solution, respectively, for 100 g and 1000 ml, and kept continuously stirring for 24 h. After the liquid is stirred and then centrifuged, the sample is washed out with distilled water until the washings are free from any free ions. This product is synthesized by drying the obtained slurry in the electric oven for six hours at 450 °C^29^. To prepare the Fe-ZSM5 zeolite catalyst, use 1000 ml of 0.5 M FeCl_3_ as a solution, following the same procedure as for the "Cu-ZSM5" preparation^30^.

#### Preparation of bimetallic catalysts

Deionized water (1000 ml), was used to dissolve the 20 wt % of ceria nitrate and copper for solution preparation, and then it was mixed with 5 g of ZSM5. After, the obtained mixture is magnetically stirred for 24 h at 8 °C. Subsequently, the mixtures are repeatedly washed using deionized water to neutral range pH. The obtained consolidated material was then simply air dried and underwent a heat treatment for 4 h at 600 °C^31^. In preparation of "Cu–Ce-ZSM5", a wt.% of ceria and 20 wt.% of iron were used, following the same procedure as for the "Fe–Ce-ZSM5″ preparation^32^.

### Fly-ash and zeolites characterization

#### XRF spectroscopy analysis

**Fig. 4:**
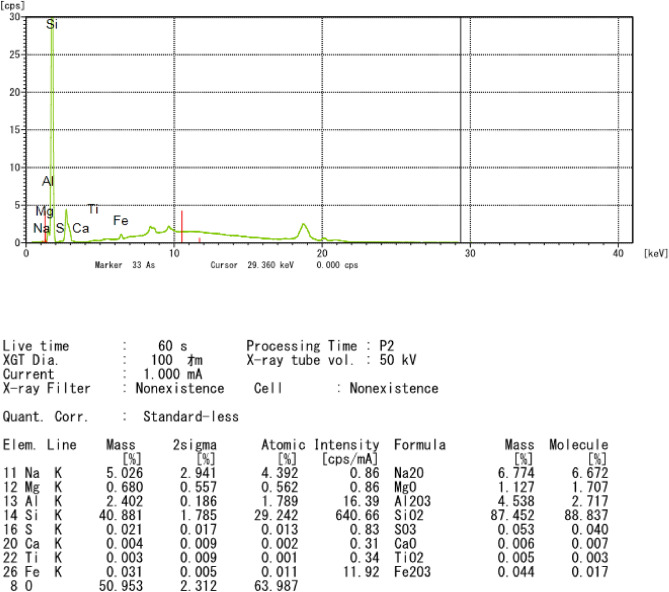
Composition of commercial zeolites (weight %).

**Fig. 5:**
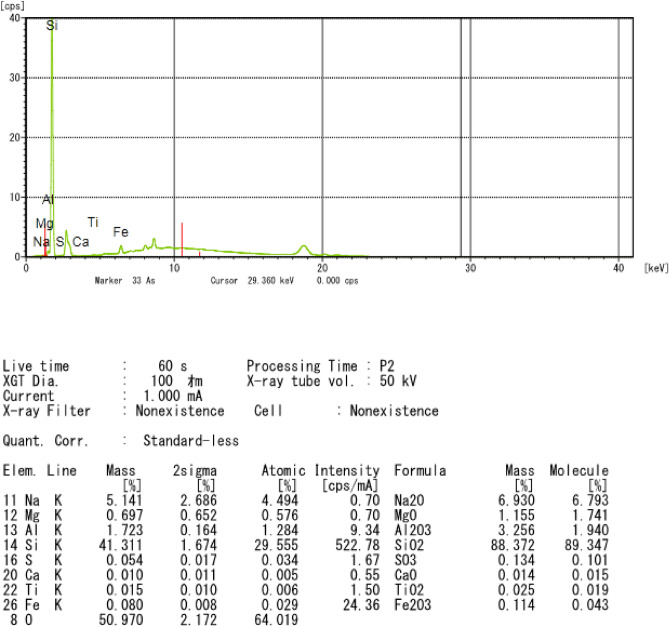
Composition of self-made zeolites (weight %).

The X-ray fluorescence (XRF) elemental compositions of the commercial ZSM-5 and the synthesized ZSM-5 (obtained based on fly ash) are shown in Figs. 4 and 5. Comparing the hydrothermal synthesis exhibited compositional differences with the original fly ash composition as demonstrated in Table 1, it is seen that there has been a major compositional change and ascertained this represented a major reorganization of the chemical structure during hydrothermal synthesis.

The commercial ZSM-5 has a standard composition comprising of SiO_2_ and Al_2_O_3_ with metal oxides having a small contribution. Conversely, the synthesized ZSM-5 exhibits a significantly large percentage of SiO_2_ and a sharp rise in the level of Na_2_O. The content of sodium oxide in the product of the synthesis varies between 0.97% and up to 14.35%. This rise in Na_2_O is not surprising and is directly caused by the alkaline synthesis environment, where the mineralizing agent is NaOH.

Na + ions are actively involved in balancing charge during the synthesis of the aluminosilicate structure. With the tetrahedral AlO_4−_ units being added to the zeolite structure, the negative framework charge is counterbalanced by further Na + ions, resulting in the increase in the Na_2_O content as observed. The increased percentage of Na_2_O in the synthesized ZSM-5 then implies that there is more framework aluminium which is necessary in catalytic ion exchange reactions.

Also, the upsurge in the content of SiO_2_ as compared to the parent fly ash indicates the successful addition of fumed silica in the preparation of the hydrothermal gel leading to the creation of a silica-laden MFI-type structure. The evidence of trace elements of Fe_2_O_3_, TiO_2_, CaO and MgO extracted during acid washing of raw fly ash is that the non-framework metal impurities were removed effectively by acid washing before the crystallization process. In general, the XRF patterns of Figs. 4 and 5 suggest that the in-house ZSM-5 has a comparable chemical composition with commercial ZSM-5 with minor differences due to the source of precursors and the conditions of the synthesis. These findings support the appropriateness of the synthesized ZSM-5 in the further metal ion-exchange and catalytic uses.

#### Scanning electron microscopy

**Fig. 6:**
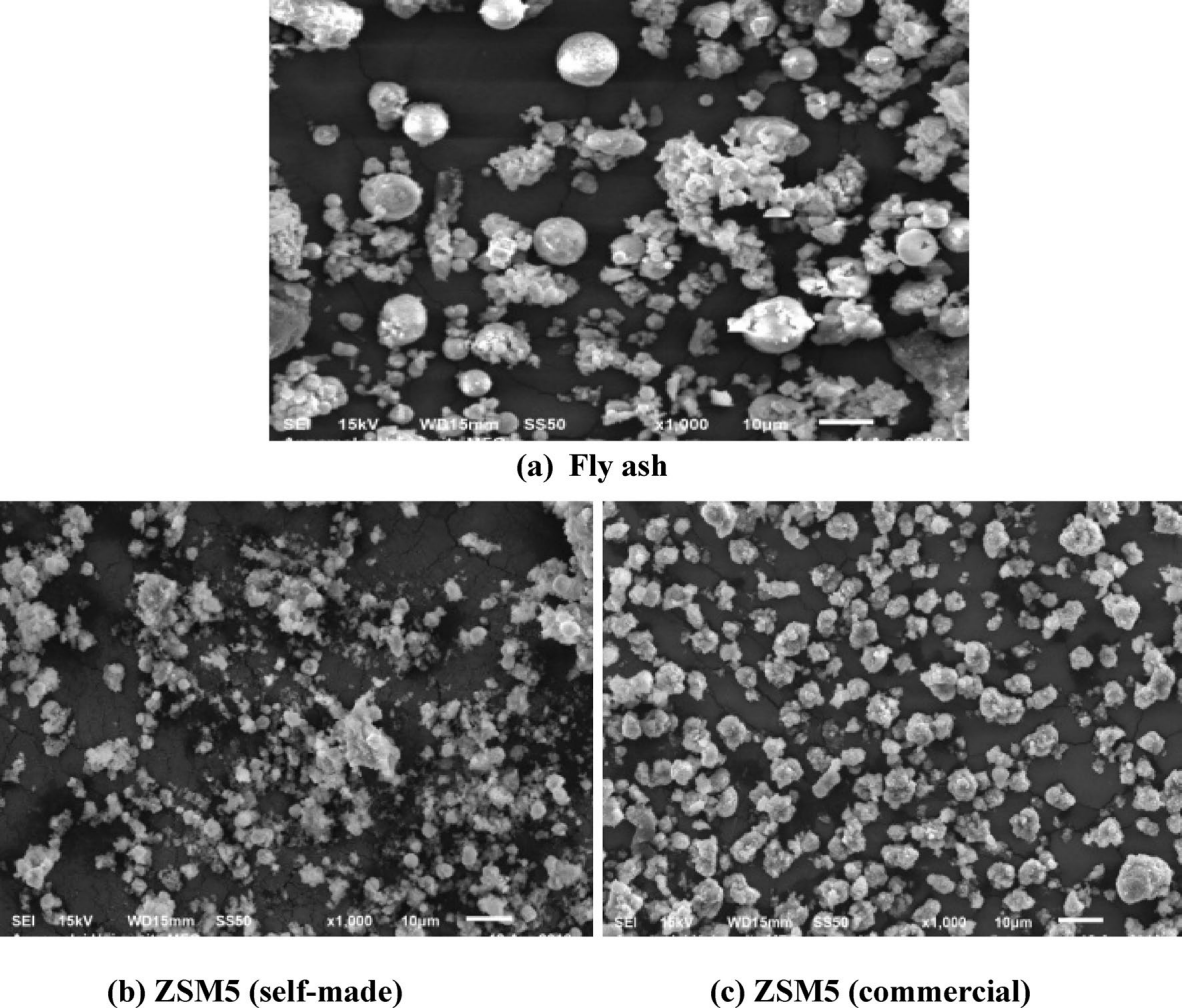
SEM image of fly-ash, self-made and commercial zeolites (parent).

A well-directed electrons beam is used to look at the ZSM5 (self-made and commercial), Cu-ZSM5 (self-made and commercial), Fe-ZSM5 (self-made and commercial), Cu–Ce-ZSM5 (self-made and commercial), Fe–Ce-ZSM5 (self-made and commercial) and raw fly ash samples in the SEM to find out about their surface structure and composition. For the analysis of samples, this study uses an electron microscope model of the JEOL-JSM 6610LV. In the first case, the upper layers of the samples undergo vapour deposition of a platinum thin layer to prevent charge entry into the samples. A voltage ranges 10–20 kV was used while taking SEM micrograph. SEM images of fly ash, and self-made and commercial ZSM5 zeolite are exhibited in Fig. 6. The figures show that fly ash yielded a high conversion of zeolite because the hydrothermally treated ZSM5-like material contained no spherical particles.

The fly ash particles exhibit low roughness due to the presence of an aluminium silicate glass layer. During this stage, the treated fly-ash surface is transformed from rough to coarse, and this indicates the stage of the formation of zeolite, also it demonstrates uniform dispersion of Cu, Fe, and Ce species on the ZSM-5 surface. The Ce-modified catalysts exhibit improved dispersion of active metal ions, which enhances the availability of catalytic active sites. It also shows that there are some minor changes (shows schistosity and has irregular forms) of the crystal structure as compared to ZSM5 (self-made) and ZSM5 (commercial).

#### X-ray diffraction (XRD)

**Fig. 7:**
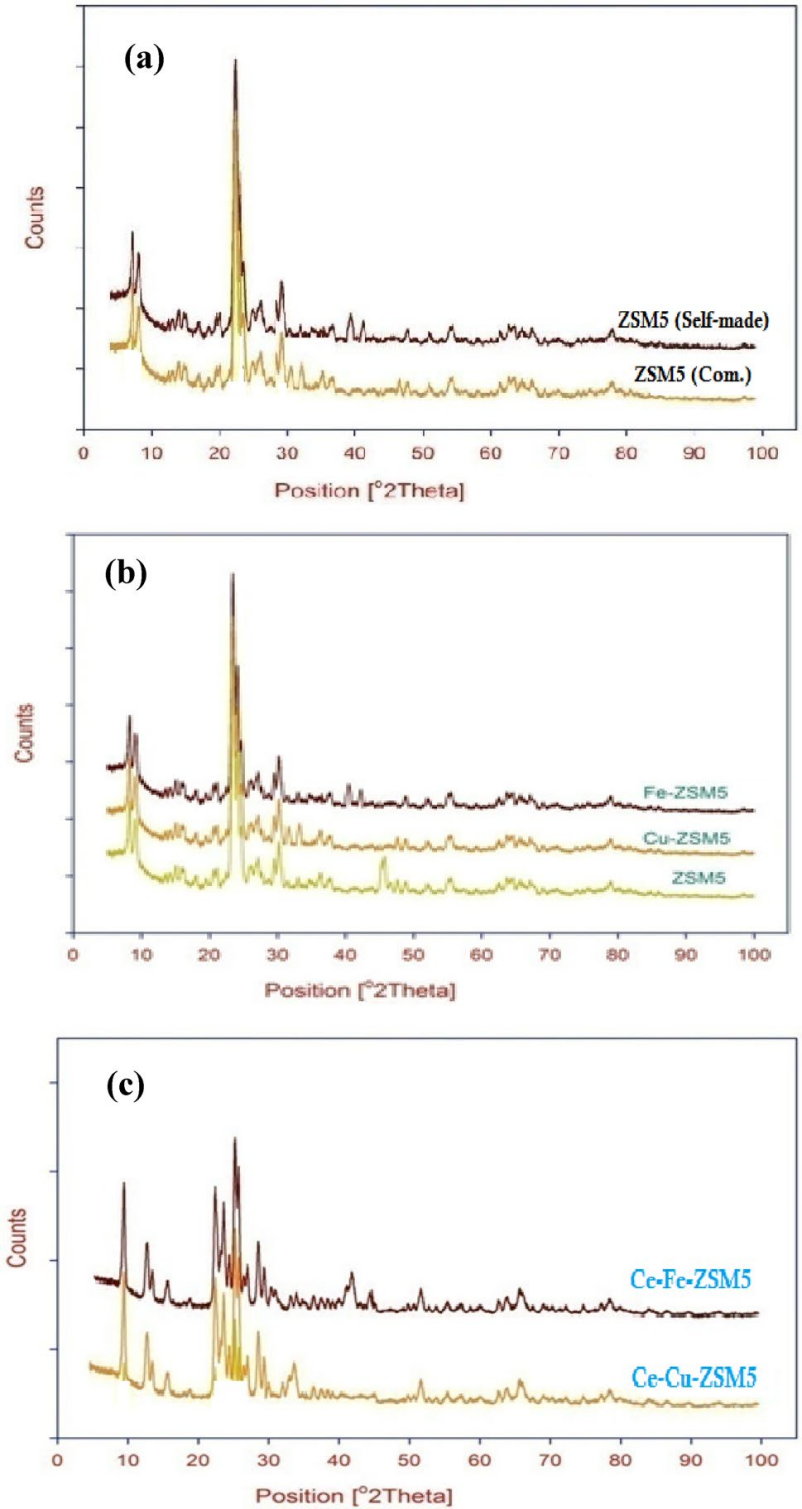
(**a**–**c**) XRD analysis of in-house zsm5 zeolite, single and double metal zeolite catalyst.

Figure 7a–c shows the X-ray diffraction (XRD) profiles of the commercial ZSM-5, in-house synthesized ZSM-5, and the metal doped zeolite catalysts (Cu-ZSM-5, Fe-ZSM-5, Cu–Ce-ZSM-5, and Fe–Ce-ZSM-5). In all patterns, the typical reflections of the MFI zeolite structure are revealed in the 2θ ranges of 7–9° and 22–25°, which proves that the synthesized sample maintains the specific crystal structure of ZSM-5. The fact that the commercial and in-house ZSM-5 patterns in Fig. 7a are similar with well-resolved peaks that are associated with the MFI topology, confirms the successful crystallization of ZSM-5 with fly-ash-derived silica-alumina precursors.

When the transition metals are introduced through the ion-exchange (Fig. 7b), all the samples maintain the basic structure of MFI structure, which means that the ion-exchange and the following thermal treatment do not damage or destroy the zeolite structure. Nevertheless, some minor yet noticeable variations in the positions and intensities of the peaks are detected, which prove the introduction of metal ions into the zeolite structure. In the case of Cu-ZSM-5, small changes in the reflections at around 2θ = 7.8° and 8.7°, and slight broadening and a decrease in symmetry at the 22.9–24.2° region are ascribed to framework distortion caused by the interaction between the Cu^2+^ and AlO_3_. The reduced strength of the peak at 29.5° indicates the presence of highly dispersed Cu species in the channels of the zeolite, which is in accordance with the literature reports on copper-exchanged ZSM-5^33^.

Equally, Fe-ZSM-5 demonstrates slight changes in the reflections at 2θ = 8° and 9° which is a coordination of Fe^3+^ with tetrahedral aluminium sites. The increased or modified peaks at 23.3°, 24.1° and 24.6° ascertain the existence of iron species. A broadened feature at an approximate of 30° indicates the potential existence of nano-sized, highly dispersed FeO_x_ domains, but the lack of large crystalline iron oxide peaks confirms that Fe is mostly included within the zeolite microstructure, as opposed to the occurrence of discrete oxide domains^34^. Although these are minor structural changes, they are significant because they are directly correlated with catalytic redox behavior, and these changes affect NO_x_ adsorption and SCR activity.

The XRD patterns of the two bimetallic catalysts (Cu–Ce-ZSM-5 and Fe–Ce-ZSM-5) are shown in Fig. 7c. The two samples both have the typical MFI reflections, which validate the claim that the addition of cerium does not interfere with the zeolite structure. There is a noticeable reduction in the intensity of the peaks of both bimetallic samples indicating a marginal loss of crystallinity, which is common when cerium is incorporated into aluminosilicate structures as a result of the partial occupation of exchange sites and enhanced lattice strain^35–41^. Notably, there are no clear crystalline peaks associated with CeO_2_, CuO or FeO_x_ phases implying that the cerium and the major transition metals are highly dispersed either in the zeolite channels or in the framework positions. This large dispersion is beneficial to catalytic use, as it increases oxygen storage ability and redox cycling.

The general diffraction patterns testify to the fact that the synthesized mono- and bimetal-doped ZSM-5 retain a strong MFI crystalline structure with minor and carefully manipulated structural distortions. The slight changes in the positions of the peaks and the differences in the intensities in Fig. 7a–c are the obvious signs of the successful incorporation of the metals. These structural changes are directly related to better catalytic performance, particularly, better NO_x_ adsorption, redox activity, and thermal stability. Therefore, the XRD study confirms all catalysts retain the characteristic MFI framework structure of ZSM-5 after metal incorporation, indicating that the ion-exchange and impregnation processes do not destroy the zeolite framework. Nitrogen adsorption–desorption results show slight reductions in surface area and pore volume after metal loading, confirming successful incorporation of active species into the zeolite channels. So, that the fly-ash-derived ZSM-5 is a structurally sound substance that can serve as an active catalyst support in diesel exhaust treatment systems. Also X-ray diffraction (XRD) analysis confirms that.

#### Performance data for the catalysts before and after the test

The catalytic activity was assessed through continuous NO_x_ reduction tests under identical operating conditions before and after the durability test. All catalysts showed only minor decreases in NO_x_ conversion, indicating good structural stability. Specifically, Cu-ZSM-5 and Fe-ZSM-5 exhibited a slight reduction in conversion efficiency due to partial sintering and minor loss of active sites, whereas Ce-modified catalysts showed better stability with negligible performance loss. The improved stability of Ce–Cu-ZSM-5 and Ce–Fe-ZSM-5 is attributed to enhanced metal dispersion and oxygen storage capacity provided by cerium, which helps maintain active redox cycles during prolonged operation. The performance comparison of the all catalysts before and after the tests details were listout the in Table 2.

**Table 2:** Performance Comparison of Catalysts Before and After the tests.

Catalyst	NO_x_ conversion before test (%)	NOx conversion after test (%)	Activity loss (%)	BET surface area change (%)	Structural stability (XRD)	Cycling stability	Key observation
Cu-ZSM-5	92–95	88–91	3–5	− 4 to − 6	Framework retained with slight intensity reduction	Good	Minor deactivation due to partial sintering
Fe-ZSM-5	88–92	84–89	4–6	− 5 to − 7	No structural collapse observed	Good	Moderate loss of active sites
Ce-Cu-ZSM-5	94–97	92–95	1–3	− 2 to − 3	Highly stable crystalline structure	Excellent	Highest low-temperature stability
Ce-Fe-ZSM-5	91–95	89–93	2–3	− 2 to − 4	Stable framework with minimal change	Excellent	Broad temperature window with strong durability

#### Cordierite monoliths

**Fig. 8:**
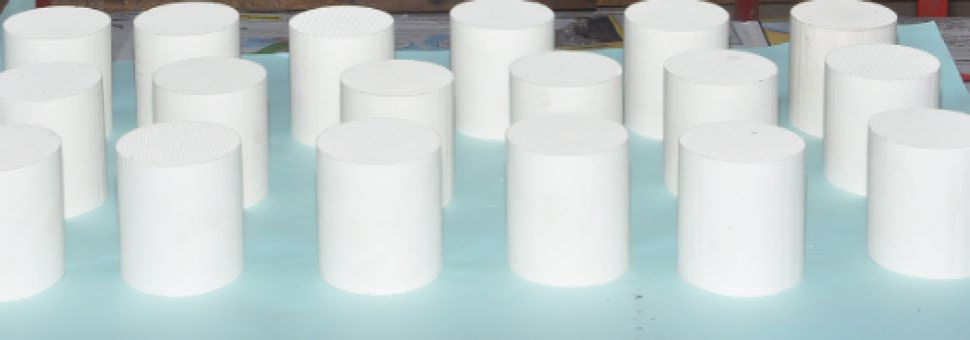
Blank monoliths.

The test engine had needs of a honeycomb monolith of cordierite honeycombs which were sourced commercially to suit the dimensional and flow necessities. The monoliths were a cylindrical design with a diameter of 70 mm and a length of 70 mm and housed 400 CPSI which is a common arrangement in diesel exhaust after-treatment systems. The dense cell arrangement provides sufficient geometric surface area to deposit catalyst yet low back-pressure at which the engine is operating. The photographic image of the blank, uncoated cordierite monoliths as the substrates on which the wash-coating was performed is demonstrated in Fig. 8.

#### Wash coating

Blank monoliths received from Bocent Ceramics Company Limited, China. The slurry was made by adding and dissolving Cu-ZSM5 zeolite (20 wt.%), and colloidal silica (2 wt.%) in 1000 ml of water. This duration was also given to each set of cordierite monoliths which were put in the slurry. Air was blown through the surfaces of the monoliths for about five seconds (as pre-timed) with forced compressed air directed on to the pathways embedded within the monoliths to remove excessive slurry. Thereafter, this monolith member underwent a heat process at 130 °C for 2 h. The monoliths were sequentially dipped into the wash coat and then dried until the substrates had the required amount of cordierite monolith wash coat (15% wt.) on them. In the end, the cordierite monolith uses Cu-ZSM5 zeolite, which was treated at 500 °C for five hours to remove organic moisture from the sample^42–45^.

Their preparation was done in a similar manner to the other zeolite containing cordierite monoliths. The wash coated monolith substrate underwent ultrasound by soaking them in an acetone inside a glass vessel and then in an ultrasonic bath for one hour at 25 °C. Subsequently, the sample is dried for two hours at 120 °C. The weight of the monolith both before and after the ultrasound treatment is measured. The weight of a wash-coated monolith sample before undergoing adherence test was 192.1 g (Table 1). After being subjected to adherence test, the final weight of monolith was 181 gm. It is observed that there is loss by 7% in weight (of wash-coat weight). Researchers^46,47^) state that a weight loss of upto 10% is an acceptable limit to indicate a stable coating adherence. Hence in this work, the overall increasing weight after ultrasonic test is approximately 14.5% with respect to monolith weight. The amount of metal (Cu) loaded into the zeolite is around 3% which was shown in Table 3 and similar procedure were followed by the remaining catalysts.

**Table 3:** Washcoat material loading on to the monolith.

No. of dip coating	Weight of monolith after loading (gm)	Percentage loading (Weight %)	Percentage loading for each dipping (%)
1	178.1	12.6	12.6
2	185.9	17.7	5.1
3	192.1	21.5	3.8
Total			21.5

#### Fabrication of catalytic converter assembly for diesel engine

The CC housing was tailor-made to suit the dimensions of exhaust of the test diesel engine and its operating needs. Every housing was designed in such a way so that it could safely hold the wash-coated cordierite monoliths and at the same time provide the appropriate flow of gas, low amounts of leakage, and structural integrity in high-temperature exhaust conditions.

**Fig. 9:**
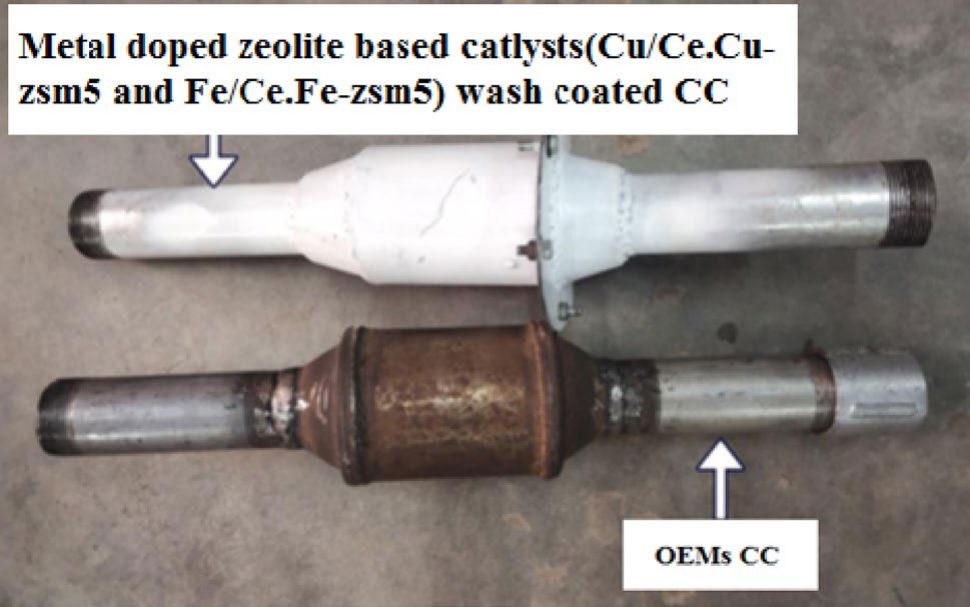
Catalytic converter photographic view.

Four units of catalytic converters were prepared, each of which had a monolith coated with one of the metal-doped zeolites catalysts produced: Fe-ZSM-5, Cu-ZSM-5, Fe–Ce-ZSM-5 and Cu–Ce-ZSM-5. These four versions provided an opportunity to compare the performance of mono-metal and bi-metal catalysts when the engine operated under the same conditions^46,47^. The catalytic converters assembled are depicted in Fig. 9, which demonstrates the metallic housing, the inlet outlet setup as well as the location of the coated monoliths in the converter body. The key parameters of the commercially available (noble metal–based catalyst containing platinum (Pt) and palladium (Pd) supported on a monolithic substrate) such as catalyst composition, geometric dimensions, cell density, and operating temperature window, as supplied by the manufacturer are shown in Table 4.

**Table 4:** Specifications of commercial catalytic converter.

Make	Tata
Country of Origin	Made in India
Substrate Material	Stainless Steel
Specific surface area	300cm^2^
Cell Density	400 Cpsi
Catalyst	Pt/Pd/Rh
Catalyst loading	1.13 g/cu.ft
Catalyst volume	1.6 lit
Material Grade	SS304
Hardness	60 HRC
Size (length × Dia.)	18 × 10 cm

## Experimental study

**Fig. 10:**
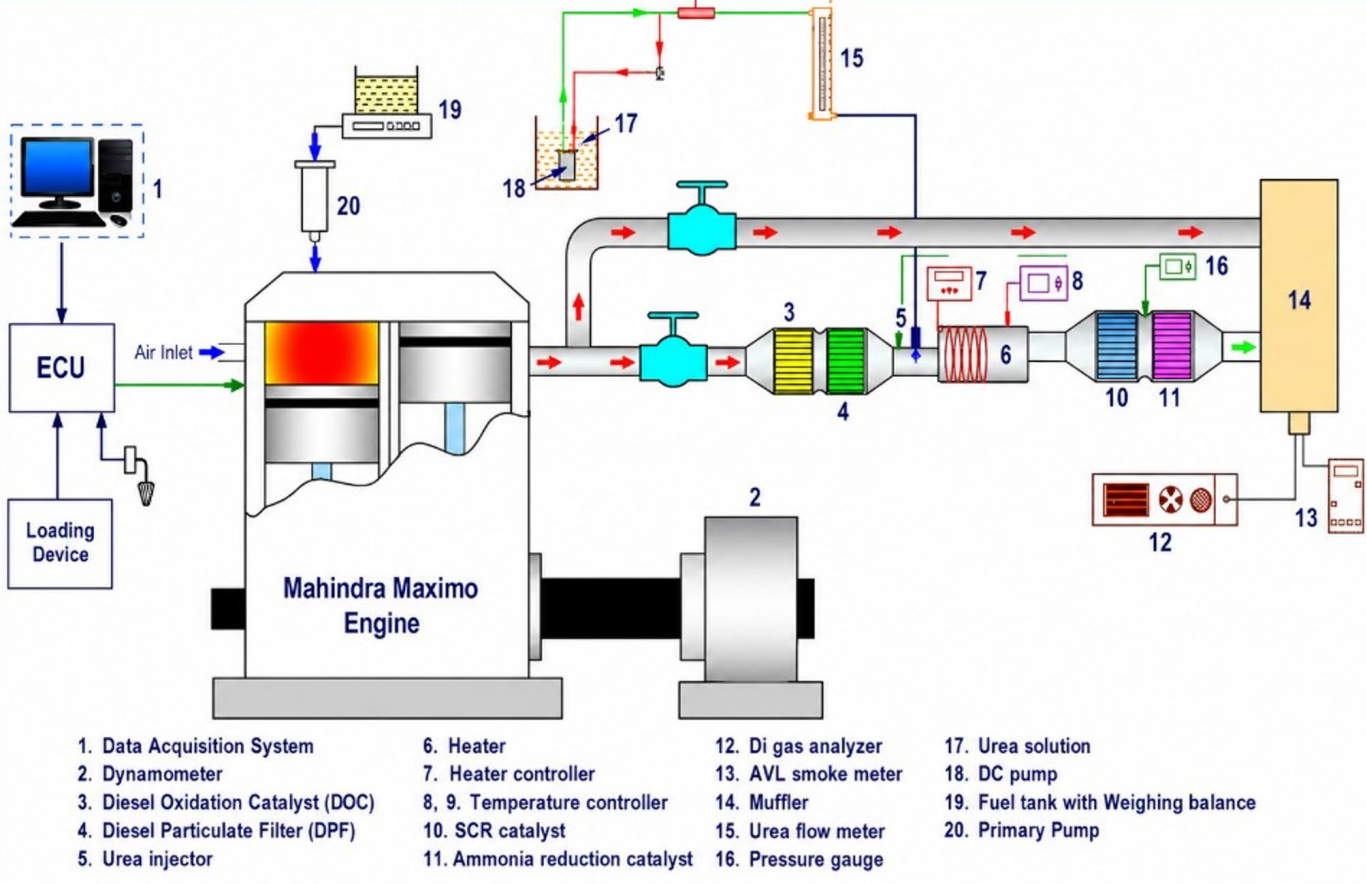
Engine setup schematic view.

**Fig. 11:**
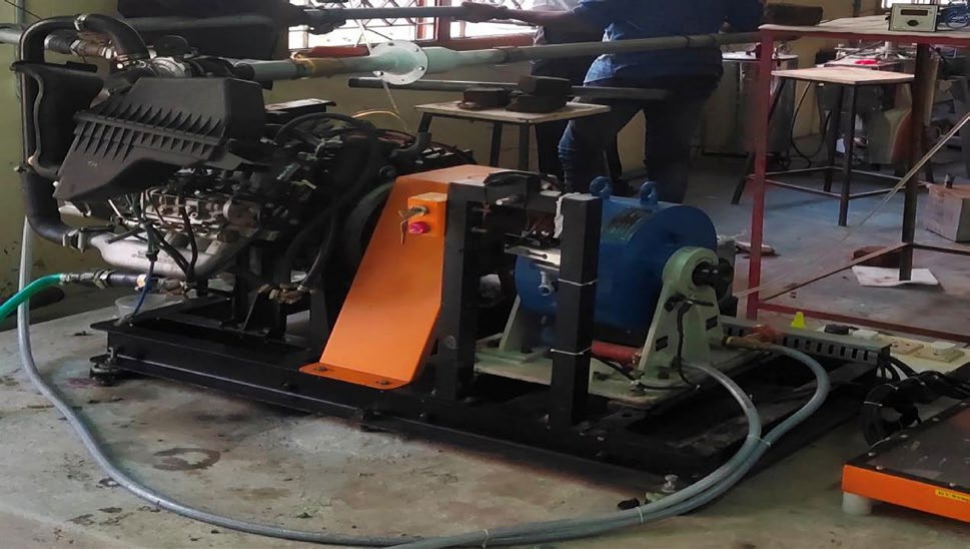
Engine setup with catalytic converter.

The test engine employed in the experiment is a Mahindra Maximo twin-cylinder, four-stroke, water-cooled CRDI diesel engine. It bewilderingly illustrates the watershed in the cycle for the flow, heating, and electrical measurements to the inserting engine load. Figures 10 and 11 depicts the schematic arrangement and photographic view of catalytic converter assembly placed on the test engine setup. Initially, the engine was permitted to operate ten to fifteen minutes for increasing the cooling water temperature until 70° centigrade is recorded with a thermal steady state condition of the engine. The engine exhaust emission (CO, HC, CO_2_, O_2_, and NO_x_) was measured by the NETEL multi gas analyzer. Chromel–Alumel thermocouples were used for exhaust gas temperature measurements, and were positioned in various locations on the converter. The tests are highlighted on the change of mechanical and thermal condition as different versions are brought intensively. First, five levels of loads (20%, 40%, 60%, and 100% load) and three levels of fuel injection pressures (FIP) (300, 600, and 900 bar) were used on the engine without a catalytic converter at the rated speed of 1500 rpm. The concentration of emissions (CO, HC, CO_2_, O_2_, and NO_x_) was recorded. Then the CC transitioned into the engine exhaust manifold. Consequently, the gases sought to pass through the converter in an axial manner. For the same period of time, the engine was highly operated under the similar operation conditions and emission levels were determined. The Specifications of Diesel engine are shown in Table 5.

**Table 5:** Specifications of Diesel engine.

Engine	
Make	Mahindra maximo
Bore	83 mm
Stroke	84 mm
Type	CRDI
Swept Volume	909 cc
Speed	2000RPM
Torque	50 Nm
Max. Load	18 kgs
Lubrication	Forced

### Space velocity calculations

In this investigation, first calculate the volumetric flow rate of air/fuel through the engine (Table 6), because space velocity (SV) is:

**Table 6:** Standard Bulk Density Values.

Catalyst	Bulk density (g/cm^2^)	Typical value used
Cu-zsm5	0.60–0.70	0.56
Fe-zsm5	0.55–0.70	0.63
Ce.Cu-zsm5	0.60–0.75	0.68
Ce.Fe-zsm5	0.60–0.72	0.66

Space Velocity(SV) = $\frac{\mathrm{V}\mathrm{o}\mathrm{l}\mathrm{u}\mathrm{m}\mathrm{e}\mathrm{t}\mathrm{r}\mathrm{i}\mathrm{c} \mathrm{f}\mathrm{l}\mathrm{o}\mathrm{w} \mathrm{r}\mathrm{a}\mathrm{t}\mathrm{e}\mathrm{R}\mathrm{e}\mathrm{a}\mathrm{c}\mathrm{t}\mathrm{o}\mathrm{r}}{\mathrm{C}\mathrm{a}\mathrm{t}\mathrm{a}\mathrm{l}\mathrm{y}\mathrm{s}\mathrm{t} \mathrm{v}\mathrm{o}\mathrm{l}\mathrm{u}\mathrm{m}\mathrm{e}}$

First, calculate the volumetric flow rate first.

*Step 1* Given data.

Displacement (Swept volume) = 909 cc = 0.909 L

Speed = 2000 rpm

Engine type = 4-stroke

*Step 2* Intake strokes per minute.

For a 4-stroke engine, one intake stroke occurs every 2 revolutions:

Intake cycles per minute = $\frac{2000}{2}=1000$

*Step 3* Volumetric flow rate.

Flow rate = Displacement × Intake cycles per minute.

 = 0.909 × 1000 = 909 L/min.

*Step 4* Convert to m^3^/hr.

909 L/min = 0.909 m3.

 = 0.909 × 60.

 = 54.54 m3/hr.

Volumetric flow rate = 54.54 m^3^/hr.

*Step 5* Calculated Catalyst volume (V).

Space Velocity (SV) = $\frac{54.54}{\mathrm{C}\mathrm{a}\mathrm{t}\mathrm{a}\mathrm{l}\mathrm{y}\mathrm{s}\mathrm{t} \mathrm{v}\mathrm{o}\mathrm{l}\mathrm{u}\mathrm{m}\mathrm{e}} \mathrm{h}\mathrm{r}-1$

Recommended catalyst mass for diesel engine and most engine exhaust catalyst studies use 30 g catalyst per experiment (Tables 7 and 8).

**Table 7:** Catalyst Volume.

Catalyst	Volume(L)	Volume (m^3^)
Cu-zsm5	0.046	4.62 × 10^–3^
Fe-zsm5	0.0476	4.76 × 10^–3^
Ce.Cu-zsm5	0.0441	4.41 × 10^–3^
Ce.Fe-zsm5	0.0455	4.45 × 10^–3^

**Table 8:** Details of Space Velocity (hr^-1^).

Space Velocity (hr^-1^) at different FIP working conditions			
Catalyst	300 bar	600 bar	900 bar
Cu-zsm5	3935	1967	1312
Fe-zsm5	3820	1910	1273
Ce.Cu-zsm5	4122	2061	1374
Ce.Fe-zsm5	3996	1998	1332

Calculated Catalyst volume (V) = $\frac{m}{\mathrm{p}}$

**For Cu-zsm5:**

Calculated Catalyst volume (V) = $\frac{30}{0.65}=46.15 cm3=0.046L=4.62\times 10-5 m3$

**For Fe-zsm5:**

Calculated Catalyst volume (V) = $\frac{30}{0.63}=47.62 cm3=0.0476L=4.76\times 10-5 m3$

**For Ce-Cu-zsm5:**

Calculated Catalyst volume (V) = $\frac{30}{0.68}=44.12 cm3=0.0441L=4.41\times 10-5 m3$

**For Fe-Cu-zsm5:**

Calculated Catalyst volume (V) = $\frac{30}{0.65}=45.45 cm3=0.0455L=4.55\times 10-5 m3$

## Results and discussions

### Oxides of nitrogen (NO_x_) emission

**Fig. 12:**
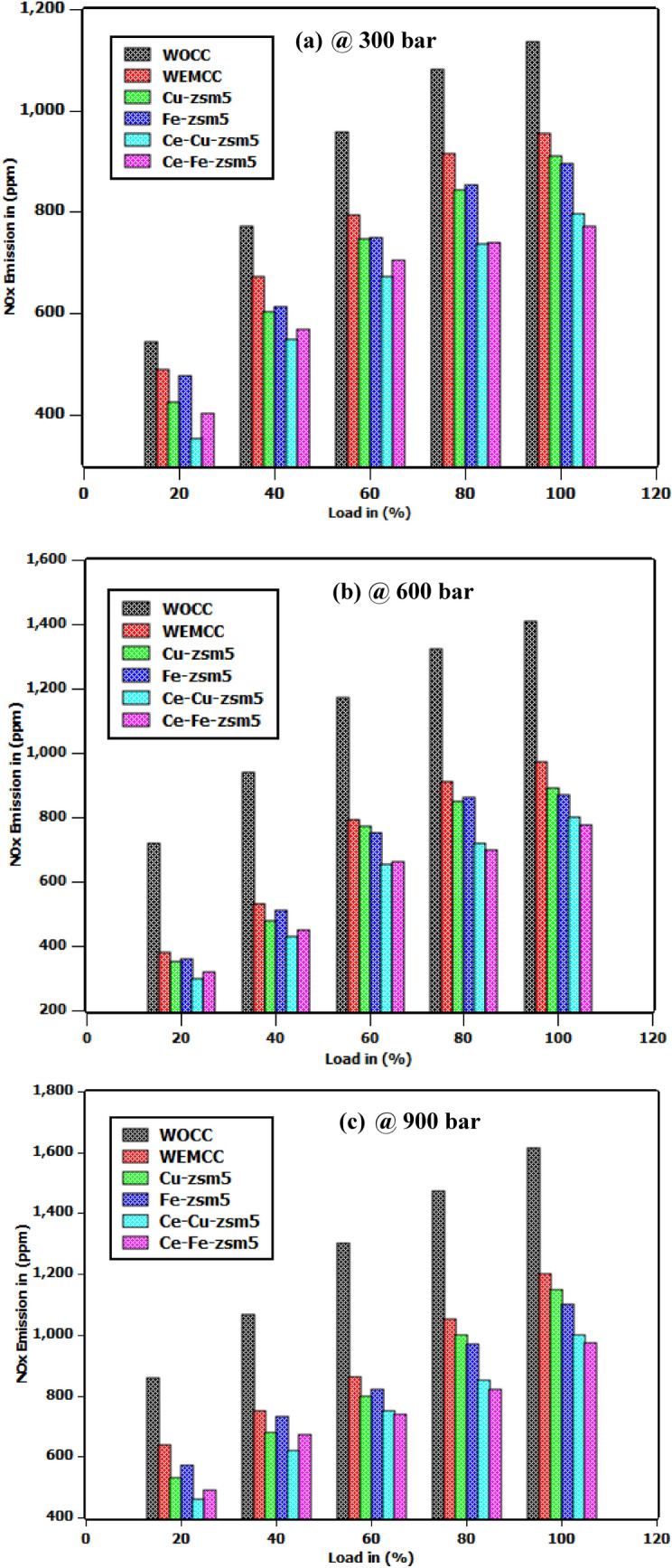
(**a–c**) Oxides of nitrogen (NO_x_) versus brake power.

Figure 12a–c indicates the change in the emission of NO_x_, in the absence of CC, with engine manufacturer catalytic converter (WEMCC), and metal doped (single and double) Zsm5 CC with regards to the different loads of the engine under different fuel injection pressures (300 bar, 600 bar, and 900 bar FIP). It is observed that the all metal doped (single and bi-metal) zeolite-based catalytic converter is able to lower the percentage of NO_x_ emission by a higher percentage as compared to the (EMCC). The metal-doped catalytic converter that was prepared with Cu and Fe lowers the amount of NO_x_ emissions not only at the idle engine loads but also at a constant decreasing under all engine load conditions. It is possible to explain the reduction in NO_x_ emission by metal-doped Zsm5 CC by the following sequential reactions of catalysis. (a) O_2_ selectively oxidized Cu + /Fe + to Cu_2_ + /Fe_2_ ± Fe _3_ + species; (b) O_2_ further oxidized the nitrogen to form a N bonded to Cu_2_ + /Fe_2_ + species; (c) the N bonded to Cu_2_ + /Fe_2_ + species was then reacted with NH_3_ to produce N_2_ and H_2_O, and regenerated Cu + /Fe +. The Cu-zsm5 CC emits the lowest quantity of NO_x_ of 20, 40, and 60% loads respectively at 80% of the loading condition and full load condition, and the Fe-zsm5 CC decreases the instability of the NO_x_ by the highest percentage in comparison to the two other conditions of loads.

Nonetheless, there is one more reason, based on the fact that when copper catalyst is used it is best operated within a constrained low temperature range of 150–350 °C. In the presence of the elements, the Fe catalyst broadens the capability of the three-way catalytic converter in high temperatures of above 450 °C to about 600 °C. Variations in brake power influence the catalytic activity that is largely determined by the temperature of the bed. As the engine shuts down, the more the brake power is increased the higher are the temperatures of the exhausts which affect the catalysts. Therefore, to obtain increased and reduced temperature activity through the enlargement of the redox window and the ultimate decrease of NO_x_ emissions, there is the use of the double metal-doped Zsm5. The dopants make the cerium more active at low temperatures and aid the conversion of Cu + /Fe + to Cu_2_ + /Fe _2_ + followed by Fe_3_ + . Redox capacity is increased by appropriate quantities of cerium additive. In order to induce the rapid conversion of Cu + to Cu_2_ + it is possible to use the simple redox reaction of Cerium: Cu_2_ + Ce_3_ + redox reaction which entail the conversion of Cu_2_ + to an enormous quantity of Cu Plus and consequently to Cu and Ce_4_ + . Despite the fact that addition of cerium enhanced the redox behaviour of Cu/Fe-Zsm5 catalysts. This was due to the fact that with Cu/Fe-Zsm5 catalyst, the cerium increased the copper valence and mobility of lattice oxygen. Thus, the addition of cerium enhances the performance of the NO_x_ emissions even further as compared it is with the Cu-doped and Fe-doped Zsm5-coated CC. Further on, it has been noted that the addition of cerium with Cu / ZSM-5 enhances the diesel selectivity towards NO by almost 95% by raising the lower and upper operating temperatures.

Lastly, a rise in FIP is likely to enhance the mono metal as well as the bi-metal doped Zsm5 CC to even a lower level of NO_x_ emission. At that, 900 bar FIP, Zsm5 CC doped with metal exhibits more NO_x_ emissions than FIPs. Since the high FIPs have a superior combustion of fuels, the exhaust gases produce more energy that increases the temperature of gases. Increase in the EGT in the exhaust gases produced also suggests an increase in the temperature of the beds thus increasing the catalyst activity in terms of increased oxidation and increased redox activity. Also, high FIP implies that light-off temperature will be achieved sooner. Further, another aspect was also noted that the Cu. Ce-zsm5 based CC unit emitted less NO_x_ at 20, 40 and 60 loads than the Fe.Ce-zsm5 based CC that minimized the NO_x_ emissions at higher loads of 80 and 100 brake powers. This is not surprising because zeolite-based ceria copper (Cu.Ce-zsm5) catalyst has a far smaller low temperature operating range of 150–400 °C. The Fe–Ce-zsm5 catalyst enhances the performance of catalytic converter repair in the temperature of 400–600 °C. Nevertheless, changes in the brake power also led to changes in the NO_x_ reduction performance of the catalyst system that is observed to be entirely dependent on the temperature of the system. The gains in the brake power resulted in gains in the exhaust gas temperature at the same time resulting in improved performance of the catalyst beds.

### Carbon monoxide (CO) emission

**Fig. 13:**
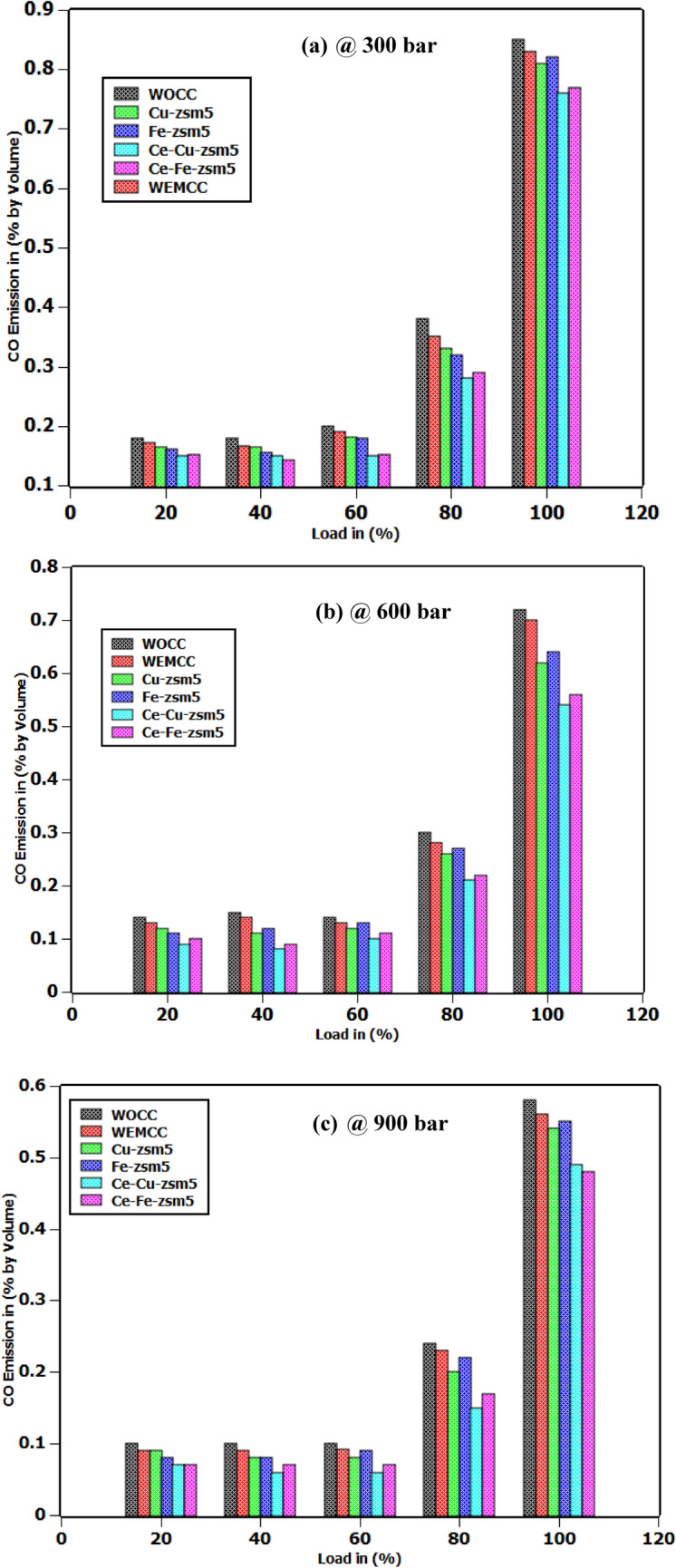
(**a–c**) Carbon monoxide (CO) versus brake power.

Figure 13a–c demonstrate the change in carbon monoxide (CO) emission with brake power at the three fuel injection pressures (300, 600 and 900 bar). The metal-doped ZSM-5 catalytic converters exhibit a low level of CO emission under all operating conditions, as compared to the engine without catalytic converter as well as the commercial catalytic converter (EMCC). The average CO conversion efficiency is nearly 80% in the entire load range and the maximum conversion is about 84% at about 80% engine load.

The main sources of CO emissions are unfinished combustion and hot spots of fuel in the cylinder. Despite the fact that the diesel engines typically work in lean-burn conditions, the difference in fuel–air mixing (especially at high loads) may cause inadequate oxygen supply in the rapid burn phase. This will lead to incomplete oxidation of fuel and production of CO. The fact that viscosity of the fuel rises with the increase in injection pressure and loads also adds to the provisional inhomogeneity of the mixtures, hence raising the CO formation.

The addition of metal-doped ZSM-5 catalysts increases the CO oxidation because the redox activity of the Cu and Fe species is strong and thus, the transformation of CO to CO_2_ occurs even at lower temperatures of exhaust. The following reaction mechanism is involved in the catalytic oxidation process:

$${\text{CO }} + \, \raise.5ex\hbox{$\scriptstyle 1$}\kern-.1em/ \kern-.15em\lower.25ex\hbox{$\scriptstyle 2$} {\mathrm{O}}_{2} \, \to {\text{ CO}}_{2}$$

Furthermore, oxidation reaction that takes place on the surface of the catalyst may also transform NO to NO_2_ under excess oxygen conditions by the following reaction:

$${\text{2NO }} + {\text{ O}}_{2} \, \to {\text{ 2NO}}_{2}$$

The enhanced CO conversion in the Cu–Ce and Fe–Ce formulations is due to high oxygen storage and release potential of cerium that helps to sustain active oxygen on the catalyst surface. This guarantees proper oxidation even in the intermittent oxygen-deficient situations. Generally, the tendencies in Fig. 13a–c indicate that the synthesized mono- and bimetal-doped ZSM-5 catalysts have a great impact in decreasing CO emissions at all working loads as well as at various fuel injection pressures.

### Hydrocarbon (HC) emission

**Fig. 14:**
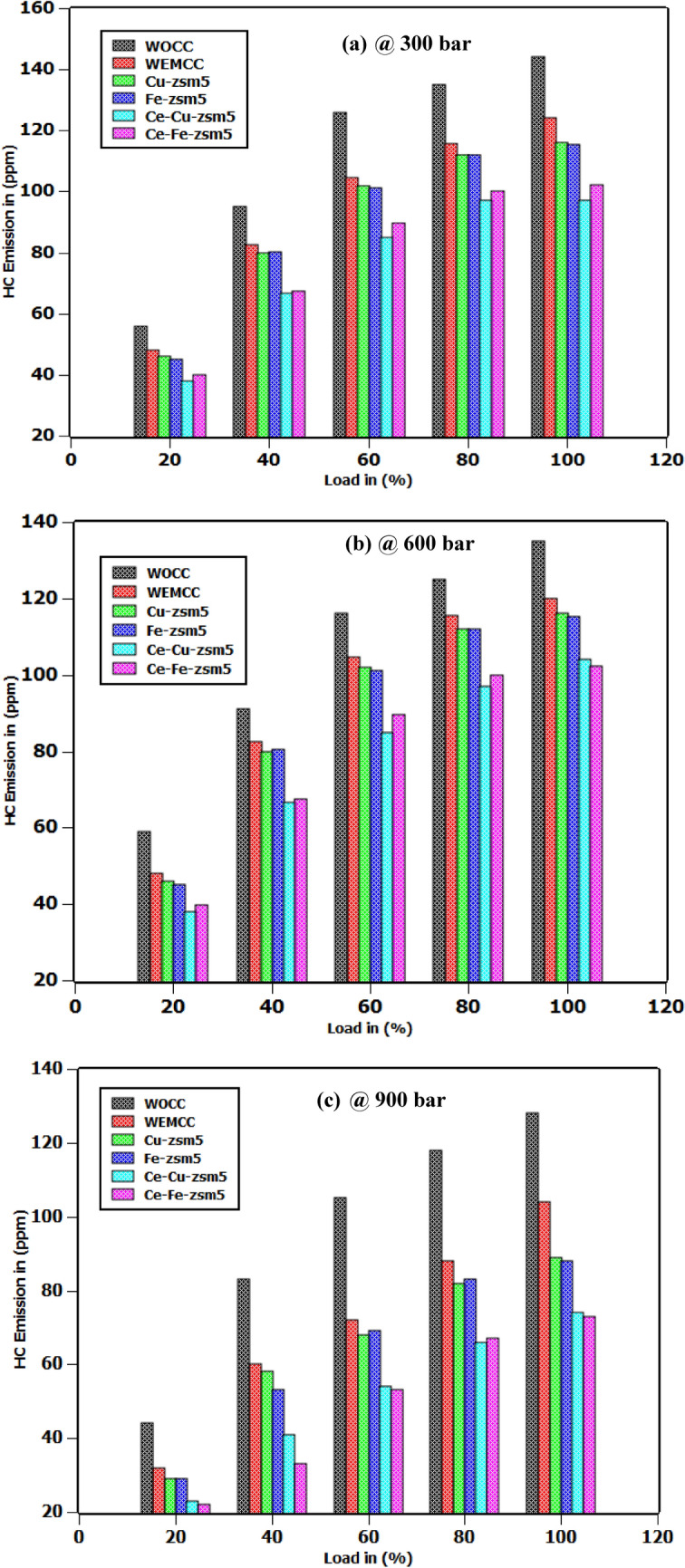
(**a–c**) Hydrocarbon (HC) emission versus brake power.

Figures 14a–c show the change of unburned hydrocarbon (HC) emissions with brake power at fuel injection pressure of 300, 600 and 900 bar. The metal-doped ZSM-5 catalytic converters have shown considerably lower HC emissions than the engine that does not have a catalytic converter and the commercial catalytic converter (EMCC) in all operating loads. Its overall HC conversion efficiency is found to be around 82% and its peak is at around 85% at about 80% brake load.

The major causes of HC emissions include incomplete combustion, wetting of the wall, incomplete atomization of fuel and the existence of locally rich regions in the combustion chamber. Even though diesel engines are generally run in a lean world state, the augmented brake force triggers augmented amounts of fuel injection and reduced ignition delays, which augment the chances of heterogeneous mixtures of fuel and air and incomplete combustion. Such conditions encourage the escape of the unburned hydrocarbons to the exhaust.

The enhanced HC reduction of the Cu- and Fe-based ZSM-5 catalysts may be explained by high oxidation capacity of transition metal ions and the acidic microporous structure of ZSM-5 framework. The active sites of the metals facilitate the oxidation of the unburned hydrocarbons to CO_2_ and H_2_O and the zeolite channels facilitate the adsorption of hydrocarbons, which allows surface reactions even at moderate exhaust temperatures. Cerium addition of the bimetallic catalysts (Cu–Ce-ZSM-5 and Fe–Ce-ZSM-5) also increases the HC conversion because of its oxygen adsorption–desorption capability, which stabilizes oxidation reactions during temporary fluctuations in oxygen concentration.

The general pattern of behavior of the synthesized metal-doped zeolite-based catalyst converters, as shown in Fig. 14a–c), indicates that the converters largely reduce HC emissions at all loads and injection pressures with the most significant reduction being recorded at mid-to-high brake loads where the exhaust temperature is favorable to catalyst activity.

### Brake Thermal Efficiency (BTE)

**Fig. 15:**
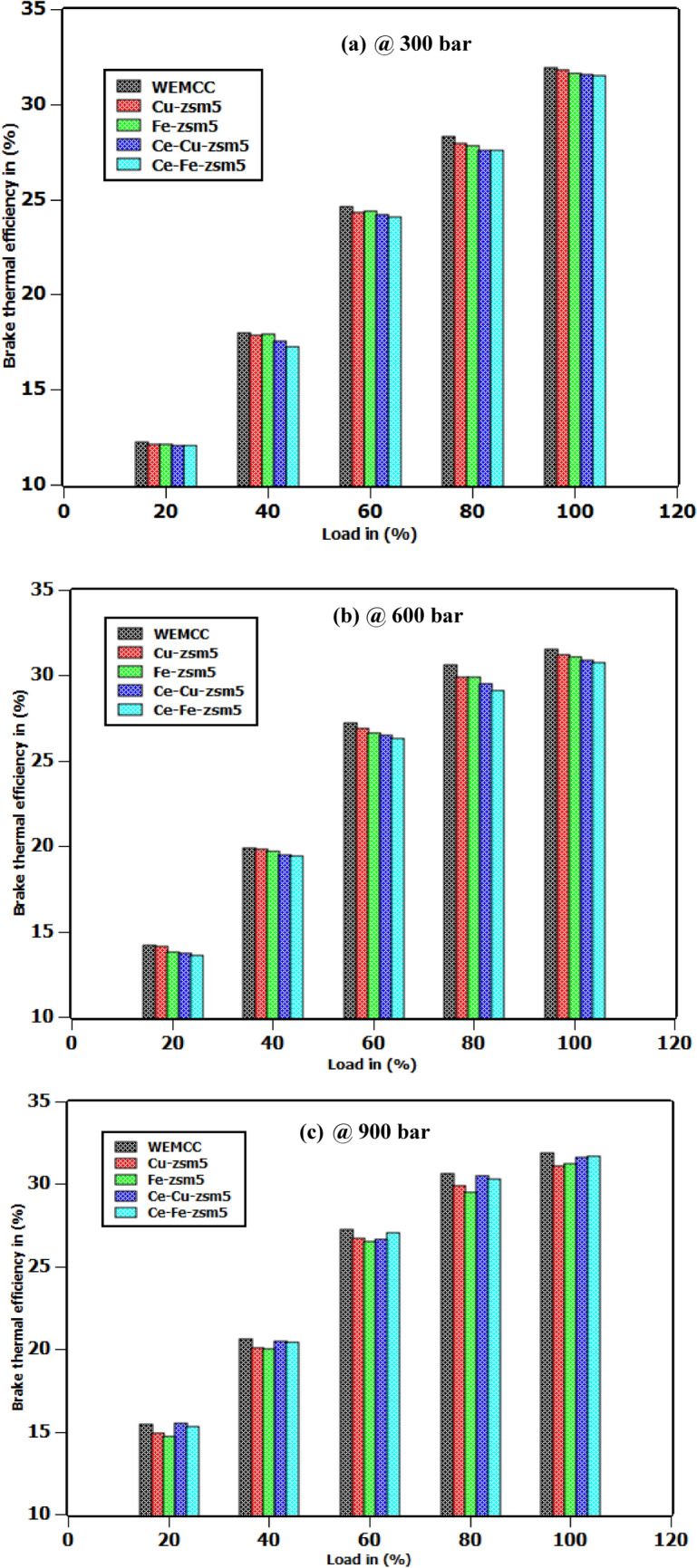
(**a–c**) Brake thermal efficiency versus brake power.

Figure 15a–c represents the change in BTE with brake power at a fuel injection pressure of 300, 600, and 900 bar. In all of the operating conditions, the inclusion of zeolite-based catalytic converters leads to only slight variations in BTE, which implies that the after-treatment system brings with it only slight performance penalties. The BTE values of all the catalyst configurations are almost similar at lower loads compared to the baseline engine, and a minor difference is caused by the difference in exhaust back-pressure. Single-metal catalysts (Cu-ZSM-5 and Fe-ZSM-5) tend to have a somewhat higher BTE than the bimetallic catalysts (Cu–Ce-ZSM-5 and Fe–Ce-ZSM-5) mainly because single-metal catalysts have lower wash-coat mass and less exhaust flow resistance, which increases in minimizing back-pressure.

The highest thermal efficiencies are obtained at the peak injection pressure of 900 bar, and the full-load BTE of Fe-ZSM-5 is about 31.20% and 30.50% and Cu-ZSM-5 and 31.60% respectively, and combustion efficiency. These values are similar or a little higher than the performance of the commercial catalytic converter (EMCC) and still similar to the performance of the diesel operation without a catalytic converter. Even though bimetallic catalysts have reduced BTE because of increased back-pressure, the difference is insignificant and compared to the fact that they have better ability to control emissions. On the whole, Fig. 15a–c prove that the zeolite-based catalytic converters synthesized have a stable thermal efficiency and do not provide any serious negative impact to the engine performance.

## Conclusion

The present work developed a zeolite-based catalytic converter that was made with low cost and utilization of coal fly ash as well as tested the same under actual diesel engine exhaust conditions. The key conclusions of this study are as follows:

1. The alkaline hydrothermal method was found to turn coal fly ash into a ZSM-5 zeolite-like product, which is an efficient and sustainable method of re-using industrial waste.
2. SEM, XRF, and XRD characterization established that the synthesized ZSM-5 has structural and compositional characteristics that are almost identical to commercial ZSM-5, which confirms its applicability as a catalyst support.
3. Effective ion-exchange incorporation of transition metals (Cu and Fe) and bimetal (Cu–Ce and Fe–Ce) into the zeolite did not change the MFI crystalline structure.
4. Bimetallic catalysts (Cu–Ce-ZSM-5 and Fe–Ce-ZSM-5) were found to have better catalytic activity than monometallic ones, being better redox behavers over the entire exhaust temperature range.
5. The synthesized catalysts of Cu–Ce-ZSM-5 (71%) and Fe–Ce-ZSM-5 (69%) demonstrated the highest efficiencies of conversion of NO_x_ at full load conditions as compared to the commercial catalytic converter (65%).
6. The CO and HC emissions of all the synthesized catalysts significantly dropped when all the engine loads were varied, indicating that all the catalysts had high oxidation capacity and that engine BTE remained constant.

Overall, the studied catalysts, Cu-Ce-ZSM-5 was the most effective in the low-temperature activity, which is especially applicable to the light-duty diesel engines, where the exhaust temperature varies most of the time.

## Data Availability

All data generated or analysed during this study are included in this published article.
